# Genome Skimming: A Rapid Approach to Gaining Diverse Biological Insights into Multicellular Pathogens

**DOI:** 10.1371/journal.ppat.1005713

**Published:** 2016-08-04

**Authors:** Dee R. Denver, Amanda M. V. Brown, Dana K. Howe, Amy B. Peetz, Inga A. Zasada

**Affiliations:** 1 Department of Integrative Biology, Oregon State University, Corvallis, Oregon, United States of America; 2 USDA-ARS Horticultural Crops Research Laboratory, Corvallis, Oregon, United States of America; University of Utah, UNITED STATES

## Introduction

Genomic data acquisition is now trivial for biologists. Yet, moving from millions of sequence reads to an assembled and annotated genome continues to pose a daunting challenge. The first animal genome sequenced arose from the free-living model nematode *Caenorhabditis elegans* [1]. This venture provided an unprecedented foundation for new insights into genome function and ‘omics tool development. However, the *C*. *elegans* endeavor has been tough to repeat, even with the advent of new high-throughput DNA sequencing technologies. For example, the first plant-parasitic nematode (PPN) genomes were published ten years after the *C*. *elegans* genome [2,3], and only five publication-quality PPN genomes are presently available [4–6].

**Fig 1:**
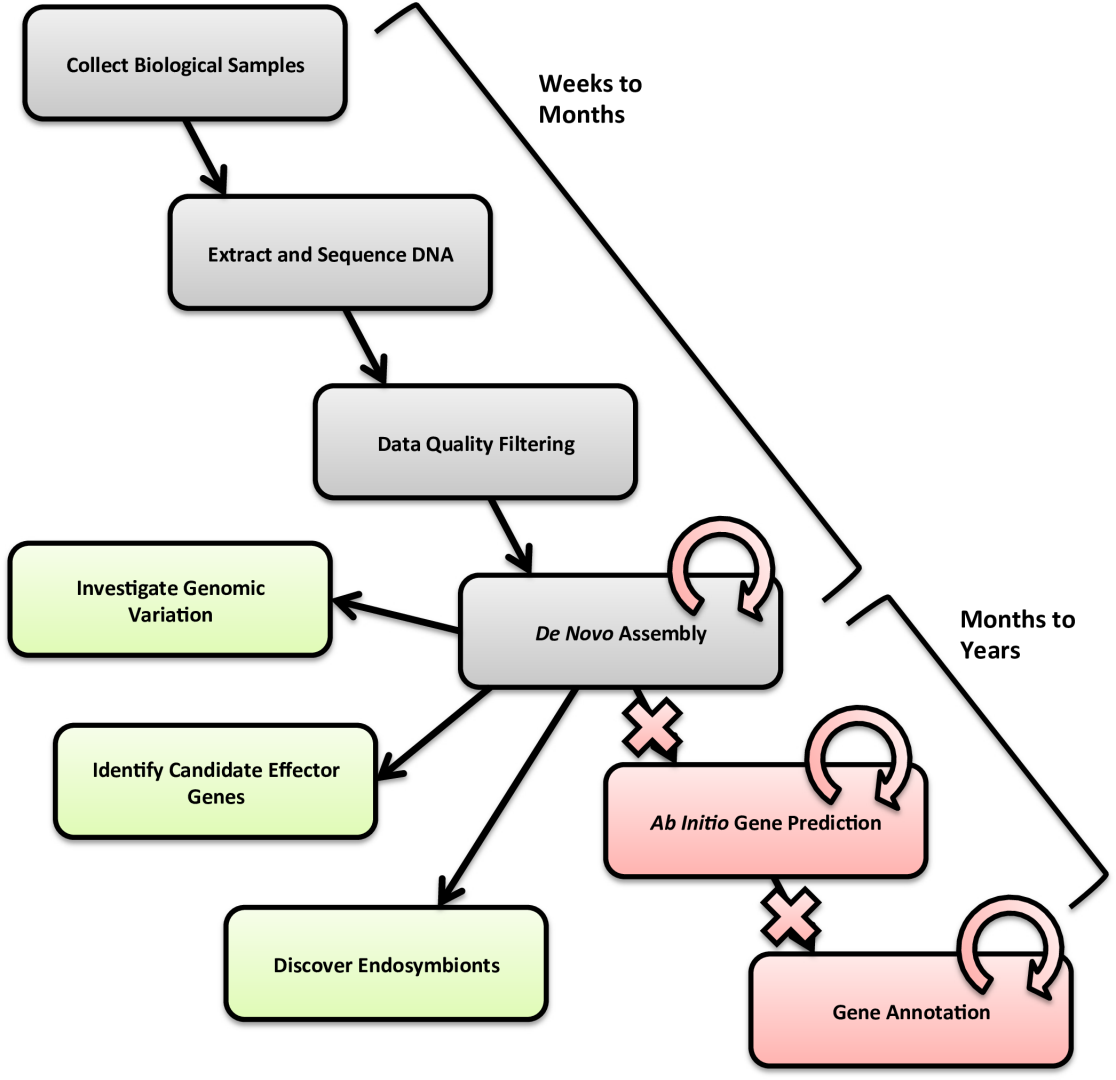
Genome skimming schematic. Boxes progressing diagonally from top left to bottom right show steps typical of conventional genome projects. Grey boxes show steps shared by genome skimming and conventional genome projects. Red boxes, arrows, and Xs show conventional genome project steps eliminated in the genome skimming approach. Green boxes show analyses specific to our genome skimming strategy.

Fig 1 overviews the course of a typical genome project. Millions of DNA sequences are initially collected in a matter of days, thanks to new DNA sequencing technologies. Early analytical phases (quality control and initial assembly) are also quick and usually straightforward. However, the subsequent computational stages (refining the assembly, gene prediction, and annotation) present significant bioinformatics bottlenecks. These lengthy in silico steps require multiple iterative stages of analysis, finally leading to a finished genome deemed “good enough” for publication. These latter stages often take years.

The term “genome skimming” was recently coined [7–9] to describe shallow sequencing approaches aiming to uncover conserved ortholog sequences for phylogenomic studies. Here, we overview a genome skimming strategy applied to six PPN species but expand the scope beyond phylogenetics and toward diverse questions relating to pathogen function and biology. We demonstrate our strategy’s utility in rapidly revealing insights and new hypotheses relating to nematode genome structure, effector genes, and endosymbionts.

## Genome Assembly Results

We applied our genome skimming strategy (Fig 1; see S1 Text) to six PPN species: *Anguina agrostis*, *Globodera ellingtonae*, *Pratylenchus neglectus*, *P*. *penetrans*, *P*. *thornei*, and *Xiphinema americanum*. Five of these species are in the “top ten” list of nematode plant pathogens [10]. Our approach begins like most genome projects by creating a single unrefined assembly for each PPN that provides a reference set of sequences for subsequent study. The lengthy downstream bioinformatics steps of typical genome projects, however, were simply not done. After completing single-pass assemblies, we examined the basic properties of the assembled contigs (Table 1). Assemblies yielded between ~10,000 and ~50,000 contigs per PPN, with average n-fold DNA sequence coverage values ranging from 7.7X to 30.4X. With an average coarse genome size estimate of 107.1 Mb and average GC content of 40.5%, these 6 PPN genome assembly patterns are consistent with known nematode genome size ranges [11,12]. We note that our smallest estimate (38.5 Mb) came from *X*. *americanum*, whose relative in the family Longidoridae, *Longidorus kuiperi*, also has a small genome size estimate of 56.5 Mb [13]. The N50 statistic, a common statistical measure for average length of a set of sequences (see S1 Text for more detail) was 8,863 bp on average for the six PPN species analyzed. Since nematode genes average ~2–3 kb in length [1,11,12], the contigs resulting from our single-pass assembly are sufficiently long to be useful database resources for BLAST [14].

**Table 1:** Genome skimming summary information and effector gene hits.

	Aa	Ge	Pn	Pp	Pt	Xa
***Genomics Summary***						
Number of nematodes	17,000	37,000	48,000	14,700	79,000	1,000
μg DNA yield	1.6	5.4	9.0	1.45	9.4	1.5
Number of reads	9,133,652	10,453,612	11,109,554	10,653,645	8,517,724	7,937,548
Bases sequenced (Mbp)	2.5	2.8	3.0	2.9	2.3	2.2
Insert size (mean +/- SD)	525 +/- 114	530 +/- 99	552 +/- 130	496 +/- 153	560 +/- 57	556 +/- 78
Maximum RAM for assembly (Gb)	60.1	57.7	60.8	94.7	53.8	57.0
Assembly time (min)	16	18	26	27	24	18
% of reads assembled	61.8	61.5	69.2	16.1	61.7	32.6
Number of contigs	35,380	18,033	13,212	37,555	47,845	31,176
Contig lengths sum (Mbp)	154.2	100.8	129.8	56.3	163.2	38.5
N50 (bp)	7,409	11,355	26,618	1,309	5,673	936
Largest contig (bp)	97,848	172,336	333,542	39,629	105,621	48,513
Average coverage	9.76	30.4	15.1	7.7	8.6	16.3
% G+C	39.0	36.7	43.9	38.5	40.0	44.6
***Effector Genes Hits***						
Annexin	+	+	+	+	+	+
β-1,4-Endoglucanase	+	+	+	+	+	+
Cellulose Binding Protein	–	+	–	–	–	–
Chorismate Mutase	–	–	–	–	+	–
Fatty Acid & Retinol Binding Protein	+	+	+	+	+	–
Peroxiredoxin	+	+	+	+	+	+
Pectate Lyase	–	–	+	+	+	–
SPRYSEC	–	+	–	–	–	–
Transthyretin-like Protein	+	+	+	+	+	–
Venom-like Allergen Protein	+	+	+	+	+	–

## Characterizing Genomic Variation

Early genome sequencing initiatives focused on model organisms such as *C*. *elegans*, in which sequenced DNA came from highly inbred lab populations. Modern pathogen genomics, however, often requires analysis of natural populations in which numerous factors can lead to deviations from the genomic uniformity of an inbred lab culture. For example, pathogens may display population-level genetic variation, within-individual heterozygosity, and other deviations (e.g., polyploidy or interspecies hybridization). These pose potential challenges but also opportunities for discovery. Interspecies hybridization and associated genome admixture is of increasing relevance to natural parasite populations [15]. *Meloidogyne incognita*, the world’s most devastating PPN species, evolved through between-species hybridization, as evidenced by recent phylogenomic analyses and the complex ploidy state of its nuclear genome [2,16]. The extent of hybridization among PPN species, however, remains unclear.

**Fig 2:**
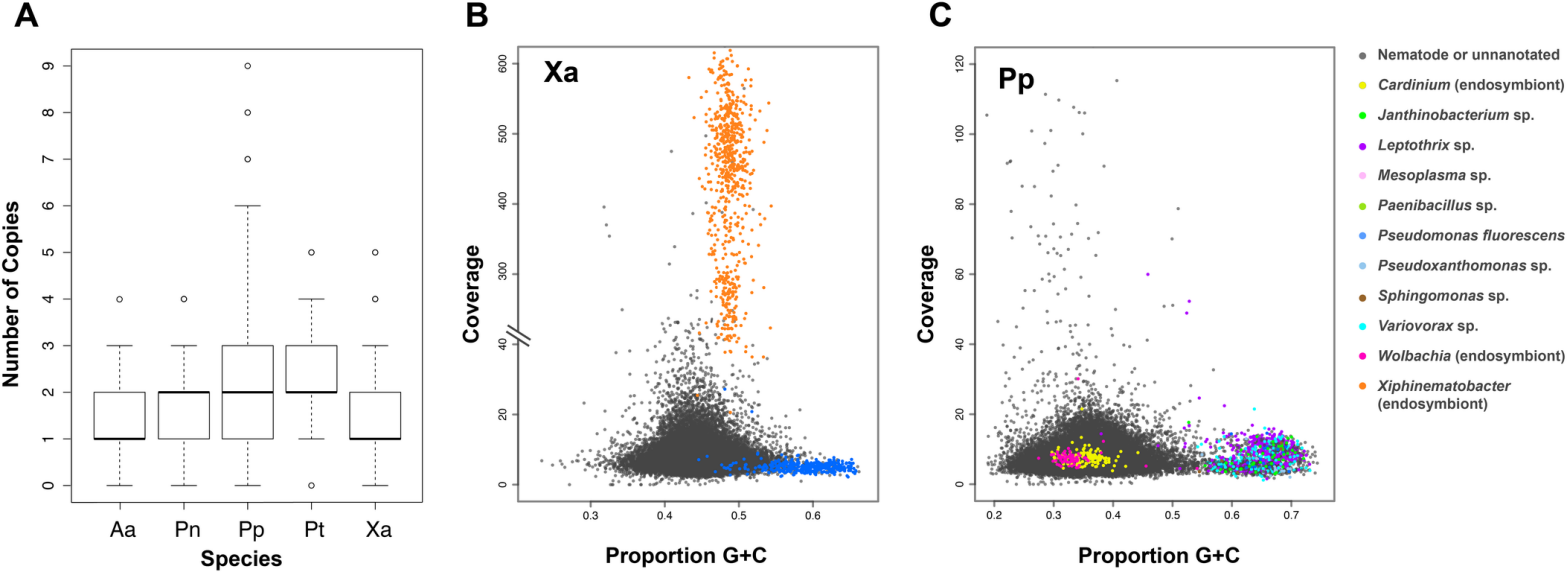
Box and blob plots. (A) Box plots reporting results for numbers of homologs detected for 65 highly conserved orthologs in 5 PPN species analyzed. Results for *G*. *ellingtonae* are not included because this species was found to encode a single homolog for all 65 orthologs. (B) and (C) Blob plot results for *X*. *americanum* and *P*. *penetrans*, respectively. Colors indicate BLAST matches to different species of bacteria.

We developed a simple BLASTN-based method to quickly screen for evidence of genomic variation, using a list of 65 conserved single copy orthologs found in the genomes of *C*. *elegans* and *G*. *rostochiensis* (S1 Table) and our PPN genome assemblies. *G*. *rostochiensis* orthologs were used as queries against our *G*. *ellingtonae* contig database; single hits were found for all orthologs in the latter species, suggesting a high degree of genomic uniformity in the sample sequenced for this species. For the other 5 PPN species, however, more variable results were observed (Fig 2A). The median number of orthologs was equal to 1 for 2 species (*A*. *agrostis*, *X*. *americanum*), with small variances in copy number among the 65 genes (0.56 for *A*. *agrostis*, 1.25 for *X*. *americanum*). This small variation likely reflects some small genetic variation among the nematodes sequenced and/or the occurrence of lineage-specific duplicates for some of the orthologs. The median number of orthologs detected was 2 for all 3 *Pratylenchus* species. For the *P*. *penetrans* sample, it was known that nematodes from many field populations were combined in the sample used for the Illumina run, and thus, this genomic diversity is reflected in the high variance in ortholog copy number calculated for this species (4.46). The sequenced DNA samples for *P*. *neglectus* and *P*. *thornei*, however, each came from single nematode populations. The variances for these two species (0.67 and 0.95, respectively) were similar to those calculated for *A*. *agrostis* and *X*. *americanum*. The median value of two copies per ortholog for *P*. *neglectus* and *P*. *thornei*, combined with their low variance, suggests possible tetraploidy in these species. This hypothesis is supported by cytological evidence collected nearly 50 years ago [17] suggesting tetraploidy for *P*. *neglectus* and diploidy for *P*. *penetrans*.

## Finding Effector Genes

Discovery and functional characterization of effector genes, whose products directly engage in attacks on host defenses, is a central aim of any pathogen genome project. Protein sequences for 10 effectors, well characterized in other PPN species (S2 Table), were used as TBLASTN queries to screen our PPN contig databases for homologous matches. Our search revealed 42 matches (out of 60 possible) distributed across the PPN genomes (Table 1). As expected, more hits were observed in the 5 tylenchid PPN species analyzed (ranging from 6 to 8) compared to the very distantly related *X*. *americanum*, in which only 3 hits were observed. These 3 genes (annexin, β-1,4-endoglucanase, peroxiredoxin) were found in all 5 of the other species studied; a previous study revealed evidence for an expressed endoglucanase effector in *X*. *index* [18], a congener of *X*. *americanum*. The 3 *X*. *americanum* hit e-values (averaging 7.1 E-30) and hit lengths (averaging 459 bp) were larger and shorter, respectively, compared to averages for these 3 genes in the other 5 species (1.0 E-42, 632 bp). The addition of a simple single BLAST step to our genome skimming strategy quickly revealed the presence of numerous putative effector genes in the PPN species, though follow-up experimentation and analysis remains necessary to evaluate whether or not bona fide effectors are encoded by the DNA sequences identified.

## Discovering Endosymbionts

Bacterial endosymbionts, such as *Wolbachia* spp., are well known and widespread components of diverse arthropods. Genome sequencing efforts in filarial nematode species revealed the presence of *Wolbachia*, which functions as an obligate mutualist in these pathogens of animals and humans [19,20].

We combined “Blob plot” approaches [21] with BLAST to uncover bacterial genomes associated with our PPN species. For the *X*. *americanum* analysis, evidence for its known endosymbiont *Xiphinematobacter* sp. [22] was observed as expected (Fig 2B). This genome-skimming result led to the hypothesis that the contigs in this blob constituted the *Xiphinematobacter* sp. genome. Follow-up bioinformatics, functional genomics, and fluorescence in situ hybridization (FISH) microscopy work supported this hypothesis and suggested that the endosymbiont functions as a nutritional mutualist with its nematode host [23].

A second interesting case was *P*. *penetrans*, in which 1,593 contigs matched bacterial DNA of diverse origins. Although many of these sequences contained high %GC, which were likely environmental contaminants (Fig 2C), two bacterial blobs of higher %AT were found containing contigs matching DNA of the known endosymbionts *Wolbachia* sp. and *Cardinium* sp. The only PPN previously reported to harbor *Wolbachia* is *Radopholus similis* [24]. A *P*. *penetrans* contig matched the 16S rDNA gene for *Wolbachia* in *R*. *similis* at 98% identity. Further bioinformatic and FISH work is underway to validate and build upon these initial endosymbiosis hypotheses arising from the *P*. *penetrans* genome skimming data.

## Conclusions

Genome skimming provides a rapid and affordable avenue for biological inquiry and hypothesis generation that avoids the time delays that accompany most genomic endeavors. A single-pass assembly followed by BLAST-based and other simple analyses revealed evidence for potential genomic hybridization, effector genes, and endosymbionts in the PPN genomes studied. Although genome skimming provides an effective approach to hypothesis generation, follow-up work remains necessary for hypothesis evaluation. Genome skimming alone will not suffice for biological questions requiring gene prediction and annotation (e.g., patterns of gene family expansion, instances of horizontal gene transfer). Nonetheless, our genome skimming pilot experiment provided quick and exciting biological insights and community genomic resources, essentially doubling the number of PPN species for which published genome sequence resources are available. How might our understanding of nematode pathogens change if genome skimming were applied to 600 PPN species instead of 6?

## Supporting Information

S1 Text Materials and Methods. (PDF)

S1 Table Conserved Orthologs Used in Genomic Variation Analysis. (DOCX)

S2 Table Effector Protein Sequences Used in BLAST Analysis. (DOCX)
