# Supplementary material for: Genome Skimming: A Rapid Approach to Gaining Diverse Biological Insights into Multicellular Pathogens
Source: PLoS Pathog. 2016 Aug 4;12(8):e1005713. doi: 10.1371/journal.ppat.1005713 (PMC4973915; doi:10.1371/journal.ppat.1005713)
Supplement: S1 Text — (PDF) [file ppat.1005713.s001.pdf]

Supplementary Materials and Methods

Nematode Collection and DNA Extraction

Nematodes were collected following methods previously described [1]. Mixed-stages of *Anguina agrostis* were obtained from infected bentgrass seed. *Globodera ellingtonae* eggs were obtained from cysts reared on potato in field microplots. A population of *Xiphinema americanum* originally obtained from a vineyard was cultured in soil on sudangrass in a greenhouse. Populations of *Pratylenchus neglectus* and *P. thornei* were obtained from wheat. Individuals of each species were surface sterilized in 0.01% streptomycin sulfate and cultured on sterile carrot disks. *Pratylenchus penetrans* was collected from raspberry in the field, and reared in soil on raspberry in the greenhouse. Nematodes were obtained by either directly releasing from seeds or cysts (*Anguina* and *Globodera*) or by decant-sieving from culture media (*Pratylenchus* and *Xiphinema*). After nematodes were collected for analysis (see Table 1 for numbers of nematodes collected), samples were homogenized and total DNA was isolated using a Qiagen DNeasy Blood and Tissue kit (Valencia, CA) for Illumina sequencing.

DNA Sequencing

We chose the Illumina MiSeq system for our genome skimming approach due to its fast run times and long reads. Genomic DNA libraries were prepared using the Illumina TruSeq DNA Sample Preparation Kit (San Diego, CA) following the manufacturer's instructions. DNA molecules ~650-750 bp were gel-excised following adapter ligation. MiSeq sequencing was performed for 301 X 2 cycles (paired-end). The six PPN samples were multiplexed onto a single MiSeq run using barcode-labeled adapters to distinguish each sample. Sequencing was done at

the Center for Genome Research and Biocomputing at Oregon State University. Roughly equal numbers of reads (ranging from 9.1 to 11.1 million – see Table 1) were collected for each of the six samples.

Bioinformatics

Initial Data Processing and Assembly

Sequence quality control and trimming in FastQC [2] and fastxtoolkit (http://hannonlab.cshl.edu/fastx_toolkit/) was done first, followed by *de novo* assembly of the individual sequence reads into contigs using Velvet [3]. Based on quality plots, fastxtoolkit was used to trim (4 bp from start, 125 bp from ends) then filter (removing reads < 50 bp, or < Phred score 15 for 70% of their length) the reads. In Velvet, a k-mer of just over half of the length of the trimmed reads (141 bp) was used, and paired assembly was performed with expected coverage 100, coverage cutoff 2, minimum pair count 1, and minimum contig size 600 bp, allowing insert size to be estimated automatically. N50 calculations present the size for which 50% of sequenced bases are included within a contig of this size or greater [4].

Screening for Genomic Variation

To search for intra-sample diversity, we created a database of conserved proteins with only one copy in C. elegans and *G. rostochiensis*. From these proteins, we used blastx (evalue 1e-20, hit length >100bp, similarity >60%) to identify a list of genes that were present in all 6 of our PPNs, but in only one copy in *G. ellingtonae*. From this list of 65 genes, we used a self-BLAST approach

(blastn evalue 0.1, maximum targets 2), blasting each genome to itself, and eliminating 'self-hits'. Supplementary Table 1 provides a list of the 65 conserved orthologs used as BLAST queries in our study.

Finding Effector Genes

To identify putative effector genes in the six PPN species analyzed, sequences from previously identified proteins found in secretions of different PPN species were obtained from GenBank (for accession numbers, see Supplementary Tables 2). Each protein sequence was used as a search query (TBLASTN) against each nematode genome assembly. Putative homologues were identified within a genome if BLAST E-values were 1.0×10^{-20} or smaller.

Discovering Endosymbionts

We screened for the presence of potential bacterial DNA sequences associated with the PPN genomes using a reciprocal blast approach. First assembled contigs were blasted against a database containing a wide variety of phylogenetically diverse bacterial 16S rRNA gene sequences (blastn evalue 10, maximum targets 1). Resulting contigs were blasted against the full non-redundant sequence database (nr) with default blastn values. For top blast hits that still matched bacteria, the closest full bacterial genome sequences were downloaded to make a database for plotting. Finally, all contigs were plotted in 'Blob plots' [5] (Fig 2B, C) with %GC on the x-axis and n-fold coverage on the y-axis. BLAST hit matches to bacterial genomes (evalue 1e-4, maximum targets 1) were indicated by color coding.

Data Deposition

The Illumina MiSeq data were archived with NCBI under BioProject accession number SRP068315 and in Short Read Archive under accession numbers SRR3097544, SRR3097576, SRR3097577, SRR3097580, SRR3097581 and SRR3097582.

Supplementary References

1. Ingham RE. Nematodes. Soil Science Society of America; 1994.

2. Andrews S. FastQC: A quality control tool for high throughput sequence data. In: http://www.bioinformatics.babraham.ac.uk/projects/fastqc/. 2010.

3. Zerbino DR, Birney E. Velvet: Algorithms for de novo short read assembly using de Bruijn graphs. Genome Res. 2008;18: 821–829. doi:10.1101/gr.074492.107

4. Lander ES, Linton LM, Birren B, Nusbaum C, Zody MC, Baldwin J, et al. Initial sequencing and analysis of the human genome. Nature. 2001;409: 860–921. doi:10.1038/35057062

5. Kumar S, Jones M, Koutsovoulos G, Clarke M, Blaxter M. Blobology: exploring raw genome data for contaminants, symbionts and parasites using taxon-annotated GC-coverage plots. Front Genet. 2013;4: 237. doi:10.3389/fgene.2013.00237
