# Supplementary material for: Genome Skimming: A Rapid Approach to Gaining Diverse Biological Insights into Multicellular Pathogens
Source: PLoS Pathog. 2016 Aug 4;12(8):e1005713. doi: 10.1371/journal.ppat.1005713 (PMC4973915; doi:10.1371/journal.ppat.1005713)
Supplement: S1 Table — (DOCX) [file ppat.1005713.s002.docx]

**Supplementary Table 1. *G. rostochiensis* sequences used for genomic hybridization screens**

| **Ortholog#** | **Reference Gene Name** | **KOG number** | **Full Gene Name** |
| --- | --- | --- | --- |
| 1 | GROS_g00005.t1 | KOG1373 | Transport protein Sec61, alpha subunit |
| 2 | GROS_g00149.t1 | KOG1816 | Ubiquitin fusion-degradation protein |
| 3 | GROS_g00261.t1 | KOG0180 | 20S proteasome, regulatory subunit beta type PSMB3/PUP3 |
| 4 | GROS_g00537.t1 | KOG0025 | dehydrogenase (nuclear receptor binding factor-1) |
| 5 | GROS_g01238.t1 | KOG3185 | Translation initiation factor 6 (eIF-6) |
| 6 | GROS_g01747.t1 | KOG0556 | Aspartyl-tRNA synthetase |
| 7 | GROS_g01893.t1 | KOG0815 | 60S acidic ribosomal protein P0 |
| 8 | GROS_g02531.t1 | KOG0960 | Mitochondrial processing peptidase, beta subunit, and related enzymes (insulinase superfamily) |
| 9 | GROS_g02536.t1 | KOG1498 | 26S proteasome regulatory complex, subunit RPN5/PSMD12 |
| 10 | GROS_g02854.t1 | KOG1535 | Predicted fumarylacetoacetate hydralase |
| 11 | GROS_g03233.t1 | KOG0279 | G protein beta subunit-like protein |
| 12 | GROS_g03269.t1 | KOG0559 | Dihydrolipoamide succinyltransferase (2-oxoglutarate dehydrogenase, E2 subunit) |
| 13 | GROS_g03328.t1 | KOG3459 | Small nuclear ribonucleoprotein (snRNP) Sm core protein |
| 14 | GROS_g03594.t1 | KOG3285 | Spindle assembly checkpoint protein |
| 15 | GROS_g03607.t1 | KOG0424 | Ubiquitin-protein ligase |
| 16 | GROS_g03693.t1 | KOG3405 | RNA polymerase subunit K |
| 17 | GROS_g03713.t1 | KOG2653 | 6-phosphogluconate dehydrogenase |
| 18 | GROS_g03907.t1 | KOG0466 | Translation initiation factor 2, gamma subunit (eIF-2gamma; GTPase) |
| 19 | GROS_g03910.t1 | KOG2104 | Nuclear transport factor 2 |
| 20 | GROS_g04389.t1 | KOG2519 | 5'-3' exonuclease |
| 21 | GROS_g05175.t1 | KOG0985 | Vesicle coat protein clathrin, heavy chain |
| 22 | GROS_g05408.t1 | KOG2035 | Replication factor C, subunit RFC3 |
| 23 | GROS_g05477.t1 | KOG2415 | Electron transfer flavoprotein ubiquinone oxidoreductase |
| 24 | GROS_g05491.t1 | KOG0876 | Manganese superoxide dismutase |
| 25 | GROS_g05570.t1 | KOG1272 | WD40-repeat-containing subunit of the 18S rRNA processing complex |
| 26 | GROS_g05639.t1 | KOG2770 | Aminomethyl transferase |
| 27 | GROS_g05746.t1 | KOG0820 | Ribosomal RNA adenine dimethylase |
| 28 | GROS_g05844.t1 | KOG0400 | 40S ribosomal protein S13 |
| 29 | GROS_g05981.t1 | KOG1355 | Adenylosuccinate synthase |
| 30 | GROS_g06107.t1 | KOG1795 | U5 snRNP spliceosome subunit |
| 31 | GROS_g06110.t1 | KOG2784 | Phenylalanyl-tRNA synthetase, beta subunit |
| 32 | GROS_g06217.t1 | KOG1936 | Histidyl-tRNA synthetase |
| 33 | GROS_g06340.t1 | KOG1596 | Fibrillarin and related nucleolar RNA-binding proteins |
| 34 | GROS_g06738.t1 | KOG1123 | RNA polymerase II transcription initiation/nucleotide excision repair factor TFIIH, 3'-5' helicase subunit SSL2 |
| 35 | GROS_g06756.t1 | KOG2086 | Electron transfer flavoprotein subunit beta |
| 36 | GROS_g07042.t1 | KOG0780 | Signal recognition particle, subunit Srp54 |
| 37 | GROS_g07161.t1 | KOG1534 | Putative transcription factor FET5 |
| 38 | GROS_g07549.t1 | KOG0650 | WD40 repeat nucleolar protein Bop1, involved in ribosome biogenesis |
| 39 | GROS_g07664.t1 | KOG2833 | Mevalonate pyrophosphate decarboxylase |
| 40 | GROS_g08007.t1 | KOG1556 | 26S proteasome regulatory complex, subunit RPN8/PSMD7 |
| 41 | GROS_g08120.t1 | KOG0181 | 20S proteasome, regulatory subunit alpha type PSMA2/PRE8 |
| 42 | GROS_g08412.t1 | KOG2851 | Eukaryotic-type DNA primase, catalytic (small) subunit |
| 43 | GROS_g08422.t1 | KOG4655 | U3 small nucleolar ribonucleoprotein (snoRNP) component |
| 44 | GROS_g08464.t1 | KOG0366 | Protein geranylgeranyltransferase type II, beta subunit |
| 45 | GROS_g08543.t1 | KOG2916 | Translation initiation factor 2, alpha subunit (eIF-2alpha) |
| 46 | GROS_g08559.t1 | KOG1358 | Serine palmitoyltransferase |
| 47 | GROS_g08565.t1 | KOG1781 | Small Nuclear ribonucleoprotein splicing factor |
| 48 | GROS_g08621.t1 | KOG0077 | Vesicle coat complex COPII, GTPase subunit SAR1 |
| 49 | GROS_g08842.t1 | KOG1367 | 3-phosphoglycerate kinase |
| 50 | GROS_g09185.t1 | KOG2792 | Putative cytochrome C oxidase assembly protein |
| 51 | GROS_g09210.t1 | KOG0862 | Synaptobrevin/VAMP-like protein SEC22 |
| 52 | GROS_g09290.t1 | KOG0688 | Peptide chain release factor 1 (eRF1) |
| 53 | GROS_g09484.t1 | KOG3205 | Rho GDP-dissociation inhibitor |
| 54 | GROS_g09713.t1 | KOG1350 | F0F1-type ATP synthase, beta subunit |
| 55 | GROS_g10683.t1 | KOG1770 | Translation initiation factor 1 (eIF-1/SUI1) |
| 56 | GROS_g10904.t1 | KOG2967 | Uncharacterized conserved protein |
| 57 | GROS_g10989.t1 | KOG1746 | Defender against cell death protein/oligosaccharyltransferase, epsilon subunit |
| 58 | GROS_g11214.t1 | KOG1636 | DNA polymerase delta processivity factor (proliferating cell nuclear antigen) |
| 59 | GROS_g11676.t1 | KOG1299 | Vacuolar sorting protein VPS45/Stt10 (Sec1 family) |
| 60 | GROS_g11767.t1 | KOG3189 | Phosphomannomutase |
| 61 | GROS_g11917.t1 | KOG1647 | Vacuolar H+-ATPase V1 sector, subunit D |
| 62 | GROS_g12150.t1 | KOG1643 | Triosephosphate isomerase |
| 63 | GROS_g12360.t1 | KOG3404 | G10 protein/predicted nuclear transcription regulator |
| 64 | GROS_g13443.t1 | KOG1597 | Transcription initiation factor TFIIB |
| 65 | GROS_g13685.t1 | KOG3361 | Iron binding protein involved in Fe-S cluster formation |
