# Supplementary material for: Genome Skimming: A Rapid Approach to Gaining Diverse Biological Insights into Multicellular Pathogens
Source: PLoS Pathog. 2016 Aug 4;12(8):e1005713. doi: 10.1371/journal.ppat.1005713 (PMC4973915; doi:10.1371/journal.ppat.1005713)
Supplement: S2 Table — (DOCX) [file ppat.1005713.s003.docx]

**Supplementary Table 2. Protein sequences used as BLAST queries for effector gene searches**

|  |  |  |
| --- | --- | --- |
| **Effector** | **Accession** | **Species** |
| Annexin | AAN32888 | *B. xylophilus* |
| Cellulose binding protein | A2VBB1 | *M. arenaria* |
| Chorismate mutase | AAD42163 | *M. javanica* |
| Fatty acid and retinol binding protein | CAA70477 | *G. pallida* |
| Beta-1,4-endoglucanase | Q9UA57 | *M. incognita* |
| Pectate lyase | AAQ09004 | *M. incognita* |
| SPRYSEC | CAM33004 | *G. pallida* |
| Transthyretin-like protein | CAM84510 | *R. similis* |
| Venom allergen protein | CAD60978 | *G. rostochiensis* |
